# The Role of e-Cigarette Packaging as a Health Communications Tool: A Focus Group Study With Adolescents and Adults in England and Scotland

**DOI:** 10.1093/ntr/ntae107

**Published:** 2024-06-06

**Authors:** Daniel Jones, Amber Morgan, Crawford Moodie, Georgia Alexandrou, Allison Ford, Danielle Mitchell

**Affiliations:** Institute for Social Marketing and Health, Faculty of Health Sciences and Sport, University of Stirling, Stirling, UK; Institute for Social Marketing and Health, Faculty of Health Sciences and Sport, University of Stirling, Stirling, UK; Institute for Social Marketing and Health, Faculty of Health Sciences and Sport, University of Stirling, Stirling, UK; Institute for Social Marketing and Health, Faculty of Health Sciences and Sport, University of Stirling, Stirling, UK; Institute for Social Marketing and Health, Faculty of Health Sciences and Sport, University of Stirling, Stirling, UK; Institute for Social Marketing and Health, Faculty of Health Sciences and Sport, University of Stirling, Stirling, UK

## Abstract

**Introduction:**

In the United Kingdom, e-cigarette and refill packaging must display a nicotine addiction warning. This study explored how this message is perceived, responses to alternative on-pack messages, and other options for using e-cigarette packaging to discourage youth and people who neither smoke nor use e-cigarettes while encouraging smokers to switch.

**Aims and Methods:**

Between August and September 2022, 16 focus groups (*n* = 70) were conducted to explore these topics with adolescents (*n* = 31, aged 11–17 years) and adults (*n* = 39, nonsmokers, smokers that use e-cigarettes, smokers that do not use e-cigarettes) in England and Scotland.

**Results:**

While several participants thought the current nicotine addiction warning could help increase awareness of nicotine addiction, most reported that it failed to capture attention and was not a deterrent. Alternative messages shown on packs (about harm, toxicity, wellness, litter, or relative risk) received mixed responses. Relative risk messages were perceived as most beneficial for smokers switching but also thought to potentially encourage uptake among nonsmokers. Some participants considered certain harm and toxicity messages to potentially dissuade uptake. Participants proposed several ideas to reduce the appeal of e-cigarette packaging and devices to deter youth uptake, including more prominent warnings, standardized packaging, and devices that are plain or include health messages.

**Conclusions:**

Packaging can play a crucial role in communicating product and health messages to different consumer groups. Further consideration of how packaging and labeling can meet the needs of non-nicotine users while simultaneously reaching those who may benefit from using e-cigarettes to stop smoking is warranted.

**Implications:**

While some viewed the nicotine addiction warning required on e-cigarettes and refill packaging in the United Kingdom as helpful in raising awareness of nicotine addiction, it did not resonate with most of our sample of adolescents and adults. The findings suggest that e-cigarette packaging could be better used to encourage smokers to switch to a less harmful alternative, with relative risk messages showing promise. Furthermore, strengthening on-pack messaging (eg increasing salience and rotating messages) and reducing the appeal of packaging (eg drab colors) and devices (eg including warnings) may help increase awareness of e-cigarette harms while deterring use among adolescents and nonsmokers.

## Introduction

There has been increased academic focus on e-cigarette packaging internationally.^1–4^ In the United Kingdom and most of Europe, e-cigarettes must display a nicotine addiction warning, with experts on warning labels noting that nicotine addiction warnings are informative yet insufficient for the protection of public health and may not resonate with adolescents or smokers.^5,6^ Two studies in the United Kingdom provide some support for this position. The first, conducted shortly after the nicotine addiction warning (“This product contains nicotine which is a highly addictive substance”) was legally required on e-cigarette and refill packs under the Tobacco and Related Products Regulations,^7^ found that only 10.2% of adolescents in England who had noticed warnings on packs could recall the use of the word “nicotine.”^1^ The second also found low recall of the on-pack nicotine warning, with many adolescents in England, Scotland, and Wales who had used an e-cigarette unsure whether it had contained nicotine, indicating that use was driven more by flavor than nicotine.^8^

Unlike warnings on cigarette and tobacco packaging, which aim to deter anyone from using these products, warnings on e-cigarette packs should communicate potential risks without suggesting that they are as harmful as cigarettes.^6^ Experimental survey research in the United Kingdom, where intervention participants were shown images of messages on e-cigarette packs, found that smokers who viewed a relative risk message (“Use of this product is much less harmful than smoking”) had higher intentions to quit smoking and to purchase an e-cigarette in the next month than those who viewed a message about nicotine being addictive.^9^ The relative risk message was not associated with increased intentions to use an e-cigarette among nonsmokers.^9^ Similarly, a systematic review concluded that for smokers, intentions to purchase, try, or switch to nicotine vaping products were higher when exposed to a relative risk message and lower when exposed to nicotine addiction warnings.^10^

Research suggests that communicating an array of e-cigarette harms via on-pack warnings could increase awareness of health harms (eg chemical exposure)^11^ and that expanding message themes beyond nicotine addiction could help discourage vaping among adolescents.^12,13^ Perceptions of e-cigarette warnings may differ by age, e-cigarette use, and smoking status^3,4,14–16^; therefore, it is important to consider how different groups respond and avoid unintended consequences to help promote individual and public health.^16,17^ For instance, online focus groups in the United States with youth and adults with various smoking and vaping experiences found that warnings regarding future cognitive development, memory, and mood may help deter youth e-cigarette use, whereas warnings highlighting the toxic ingredients and harms associated with e-cigarettes may not sufficiently deter youth and could, unintendedly, discourage adult smokers from switching to e-cigarettes.^16^

Standardized tobacco packaging has been shown to increase warning salience and reduce the appeal of packaging and smoking, with evidence mixed on whether it helps reduce misperceptions of harm.^18–20^ There is relatively limited research exploring other options for using e-cigarette packaging to discourage youth and people who neither smoke nor use e-cigarettes while encouraging smokers to switch. A randomized online experiment exploring youth perceptions of e-liquid packaging in England, Canada, and the United States found an association between standardized packaging and lower interest in trying products and higher health risk perceptions,^21^ with research in Great Britain suggesting that standardized e-cigarette packaging may help reduce product appeal among adolescents without reducing product appeal among adults.^22^

Most studies on warnings on e-cigarette packs are US-based or quantitative, and our understanding of the potential impact of the nicotine warning required in the United Kingdom^8^ and different warning themes^23^ is relatively limited. This qualitative research aimed to explore adolescent and adult responses to the current warning, alternative on-pack messages, and other options for using e-cigarette packaging to discourage youth and people who neither smoke nor use e-cigarettes while encouraging smokers to switch.

## Materials and Methods

### Design and Sample

Sixteen foci groups were conducted in Greater Glasgow (Scotland) and Manchester (England) between August and September 2022, with participants (*n* = 70) segmented by age, gender, social grade, and nicotine use (Table 1) to promote free-flowing discussion and explore potential demographic differences. Social grade was categorized by the occupation of the person in the household with the greatest income,^24^ with grades A, B, and C1 signifying higher- and middle-class groups and C2, D, and E working-class groups. Two of the adult groups were nonsmokers, four with smokers who do not use e-cigarettes, and four with smokers who also use e-cigarettes (dual users). It was important that the discussions drew insight from nonsmokers (who should not be encouraged to try e-cigarettes) and benefit from having smokers who use e-cigarettes and those who do not. As smoking rates are higher among more deprived groups,^25^ all smokers were in social grade C2DE. Given the low smoking prevalence,^26^ and the legal age for purchasing tobacco and e-cigarette products in the United Kingdom is 18, we neither asked about nor applied a quota on nicotine use for the six groups with adolescents (11–17-year-olds) during recruitment.

**Table 1.** Sample for Focus Groups

Group	Gender	Age	Social grade	Country	Nicotine use	Number in group
1	F	18–39	C2DE	Scotland	Smokes and uses e-cigarettes	5
2	M	18–39	C2DE	Scotland	Smokes (doesn’t use e-cigarettes)	4
3	F	40+	C2DE	Scotland	Smokes (doesn’t use e-cigarettes)	4
4	M	40+	C2DE	Scotland	Smokes and uses e-cigarettes	4
5	M	11–13	ABC1	Scotland	N/A	6
6	F	11–13	C2DE	Scotland	N/A	6
7	M	14–15	ABC1	Scotland	N/A	5
8	F	14–15	C2DE	Scotland	N/A	6
9	M	18–39	C2DE	England	Smokes and uses e-cigarettes	3
10	F	18–39	C2DE	England	Smokes (doesn’t use e-cigarettes)	4
11	M	40+	C2DE	England	Smokes (doesn’t use e-cigarettes)	3
12	F	40+	C2DE	England	Smokes and uses e-cigarettes	4
13	F	16–17	C2DE	England	N/A	4
14	F	40+	ABC1	England	Does not smoke	4
15	M	16–17	ABC1	England	N/A	4
16	M	18–39	ABC1	England	Does not smoke	4

*n* = 70, comprised of adolescents (*n* = 31, aged 11–17 years) and adults (*n* = 39).

Participants were recruited by professional market recruiters using quota sampling, with potential participants identified from existing research panels or from within their local community by a combination of door knocking of residential properties or street intercepts. Participants were screened for eligibility using recruitment questionnaires, with those eligible given an information sheet and required to provide signed consent in advance of the groups. Parental or guardian consent was also required for 11–15-year-olds. All-groups were conducted in informal venues (hotel function rooms or community centers). Aided by an iterative, semi-structured topic guide (Supplementary 1), the moderator(s) used broad, open questioning techniques to encourage participants to express themselves freely in their own words. Discussions were recorded on a digital voice-file with participant consent. All-groups were moderated by DJ, with DM and AF comoderators for the groups in Scotland. Ethical approval was granted by the General University Ethics Panel at the University of Stirling (GUEP 7691).

### Procedure

**Figure 1.**
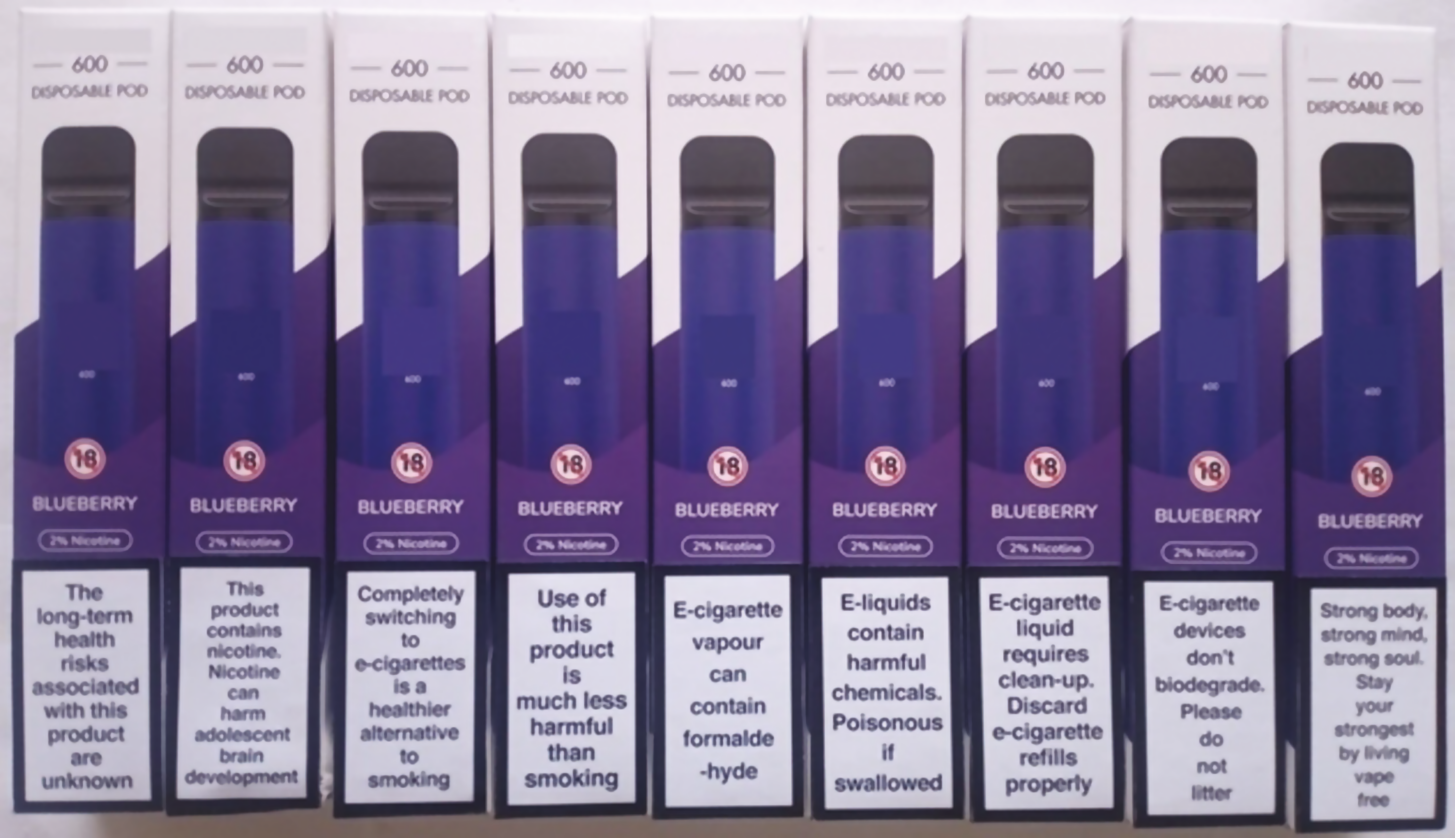
Nine alternative on-pack messages. **Notes:** Message 1—Harms: “The long-term health risks associated with this product are unknown”. Message 2—Harms: “This product contains nicotine. Nicotine can harm adolescent brain development”. Message 3—Relative risk: “Completely switching to e-cigarettes is a healthier alternative to smoking”. Message 4—Relative risk: “Use of this product is much less harmful than smoking”. Message 5—Toxicities: “e-cigarette vapor can contain formaldehyde”. Message 6—Toxicities: “e-liquids contain harmful chemicals. Poisonous if swallowed”. Message 7—Litter: “e-cigarette liquid requires clean-up. Discard e-cigarette refills properly”. Message 8—Litter: “e-cigarette devices don’t biodegrade. Please do not litter”. Message 9—Wellness: “Strong body, strong mind, strong soul. Stay your strongest by living vape free”

Similar to alcohol packaging research,^27,28^ each focus group consisted of two separate sections: e-cigarette packaging as a marketing tool (not reported here); and e-cigarette packaging as a health communications tool. Groups lasted approximately 91 min (range 84–100 min), with equal time allocated to both sections. Participants discussed their perceptions of the nicotine addiction warning on packs, alternative on-pack messaging, and other potential e-cigarette packaging regulations to reduce interest in e-cigarettes among adolescents and non-nicotine users without diminishing their potential value to people who may use e-cigarettes to quit smoking. Participants handled examples of e-cigarette packs with alternative messaging to facilitate discussion and explore their reactions. Nine alternative messages were selected from the literature, covering harms,^3,23^ relative risk,^9,29^ toxicities,^30^ litter,^30^ and wellness^31^ (Figure 1), and shown by theme in a random order. Each alternative warning covered 30% of the front and back, as is required for the nicotine addiction warning,^7^ and was shown on an Elf Bar disposable pack—the highest-selling brand in the UK^32^—for consistency. Participants were given an oral debrief, websites with information about nicotine-containing products should they wish to learn more, and £40 for participating and travel expenses.

### Analysis

Audio recordings were transcribed verbatim by a transcription company. Transcripts were reviewed for accuracy, anonymized and imported into QSR NVivo 20 for analysis. Analysis was deductive, through the topics included in the topic guide, and inductive, from participants’ responses,^33^ which has also been applied in qualitative tobacco packaging research.^34,35^ An initial coding framework was developed by reading the transcripts and discussion within the team, which was tested and refined by DJ and GA independently coding a sample of transcripts. All remaining transcripts were coded by DJ and GA independently after discrepancies had been discussed and resolved. The coded themes were then used as the categories for analysis. Three main themes and respective sub-themes were refined, labeled and interpreted by AM and discussion within the team: Responses to nicotine addictiveness warning (salience, purpose, and perceptions); perceptions of alternative messaging (believability, language and relevance, and perceived behavioral impact); alternative pack options (strengthening health messaging, standardized packaging and devices, and warnings on e-cigarettes). Representative quotations with accompanying participant features (age, gender, social grade, and nicotine use, see Table 1) are provided in the Results to illustrate findings. While few, differences between groups (eg by age and nicotine use), where these exist, are identified in the text.

## Results

### Responses to Nicotine Addictiveness Warning

#### Salience

Many participants reported that while people may look at the nicotine warning briefly, it was unlikely to capture attention or be taken seriously, eg “*I don’t think it’s severe enough for people to pay attention to it*” (16–17M, ABC1). The warning was often seen as mundane, with people desensitized to it, and had limited salience as the pack was typically discarded.

*I don’t think it’s effective, really. I think if you smoke these* [e-cigarettes] *all the time, it doesn’t enter your mind at all* (18–39M, ABC1, Non-smoker)

Several participants, mostly adolescents, thought the warning failed to stand out against the rest of the packaging, or were critical that it was contradicted by the promotional elements of the packaging.

*The only things that stand out is, like, the flavour, and the colour, and stuff. Some things that make it actually attractive. The things that don’t make it attractive don’t stand out* [Participant 3] (14–15F, C2DE)

*Okay* [Moderator]

*The only non-attractive thing that stands out is the bit that says that it contains nicotine, but other than that, it’s all just kind of stuff that would make it look good*

[*Participant* 1] (14–15F, C2DE)

#### Purpose

Most thought that the intended purpose of the warning was to inform consumers of the contents and risks associated with e-cigarettes, to discourage use, or to encourage reflection prior to purchasing.

*To warn you, so you make an informed decision . . . do I want to take this, which contains nicotine, and become addicted?* (18–39M, C2DE, Dual user)

*I think it’s supposed to be a deterrent, but I don’t think it actually deters people* (16–17F, C2DE)

*To stop people from buying them or at least think twice before purchasing it* (18–39M, ABC1, Non-smoker)

Participants often questioned the impact of the warning or were skeptical of the motives for its inclusion, viewing it as an industry “tick-box” exercise required to sell the product or protect companies from potential legal action.

*Just to cover themselves. I don’t think it’s to help anybody apart from themselves* (18–39F, C2DE, Dual user)

*I think it’s just to keep companies safe, to put them on in case they’re sued* (40+M, C2DE, Dual user)

#### Perceptions

Some participants felt that the warning was helpful and positive as it raised awareness that the device contained nicotine. However, some smokers and dual users were more likely to view the warning as pointless, claiming that it was common knowledge.

*I think it’s nice that they can put it on the product, the person knows that there’s nicotine in it* (40+F, ABC1, Non-smoker)

*It might certainly put* [off] *maybe vapers who have been non-smokers and thinking . . . maybe I shouldn’t be really doing this because I’m going to be wanting them all the time, because of the nicotine* (40+F, C2DE, Smoker)

*Everybody knows that nicotine is highly addictive* (18–39M, C2DE, Smoker)

### Perceptions of Alternative Messaging

#### Believability

Irrespective of the message shown, that they appeared on packs increased credibility, as the information was assumed to be factual and a government requirement. Several participants considered the harms and toxicities messages most believable given the belief that e-cigarettes must contain something harmful.

*You kind of trust them really . . . if you see a message on the packaging like that then, you think there’s some evidence to back it up* (40+M, C2DE, Smoker)

*I think out of all the ones we’ve seen so far, these* [harm messages] *are the ones that I would, like, endorse more than any. I think they’re probably the most compelling and, in comparison to some of the ones I’ve heard previously, probably the most honest and maybe truthful* (18–39M, C2DE, Smoker)

*It must contain harmful things, because I don’t know what else it would contain if it wasn’t. Because if it was good things in it, then it would be, like, good for your body, but it’s not good for your body* (14–15F, C2DE)

While relative risk messages (stating that e-cigarette use is less harmful or a healthier alternative to smoking) were considered believable by some participants, others contested this by pointing to a lack of credible, long-term evidence supporting the assertion that e-cigarettes are a less harmful alternative.

*Completely switching to e-cigarettes is a healthy alternative to smoking. According to who?* (40+F, C2DE, Dual user)

*It’s saying it’s less harmful, but then a lot of things are less harmful than smoking. How bad for you is it, do we know?* (18–39M, C2DE, Dual user)

#### Language and Relevance

Short, simple messages (eg “please do not litter”) were easier to understand, while the wellness message was considered *“too wordy”* (40+F, C2DE, Smoker) and was not taken seriously by most participants. Some participants felt that messages containing more demanding phrases (eg “discard e-cigarette refills properly”) provided a stronger call to action, whereas messages lacking clear instructions or further information (eg litter messages and long-term health risks) were considered vague and easy to dismiss. Messages containing words that people were unfamiliar with received a mixed response. For instance, it was thought that some people, particularly children, would not understand what formaldehyde is.

*Because it’s saying ‘please’* [do not litter]*, it’s not really got any consequences if I don’t do it, it’s just like, ‘oh please do this because it’s not very good’. Whereas that one’s* [‘discard e-cigarette refills properly’] *like ‘do it or else’* (16–17M, ABC1)

*What are you supposed to do with it? ‘It doesn’t biodegrade, don’t litter’, I mean, obviously you shouldn’t throw it on the floor but it’s* [please do not litter message] *just saying it will go to landfill, basically that, isn’t it? You can’t do anything but put it in the regular bin. No option* (18–39M, ABC1, Non-smoker)

Many participants noted that some messages were better suited to specific audiences. While most felt that the wellness message (promoting physical and emotional strength) was not useful for smokers and was aimed at nonsmokers and health-conscious people, the litter messages (encouraging people to dispose of e-cigarettes correctly) were deemed more suitable for environmentally conscious people. The adolescent brain development message was thought to be directed at young people; however, some felt that this may also target parents to discourage their own children or could be discussed in schools (eg assemblies) to highlight e-cigarette harms and deter use. The “poisonous if swallowed” message was believed to be useful for current e-cigarette users and parents to help protect children at home.

[Regarding the wellness message] *If I wasn’t already a smoker, I’d be like, “oh, Iwant to stay strong, I don’t want a vape”* (18–39F, C2DE, Dual user)

*This* [please do not litter message] *is . . . to attract the people who are conscious of the environment as opposed to being bothered by a health warning sign* (18–39M,ABC1, Non-smoker)

#### Perceived Behavioral Impact

Many participants felt that those intent on using e-cigarettes would do so regardless of the presence of a warning or message content. However, there was general agreement that the relative risk messages would be most likely to encourage smokers to switch to e-cigarettes.

*I would definitely be more inclined to buy that* [e-cigarette] *with that message* (18–39F, C2DE, Smoker)

*If I was thinking of switching to e-cigs, from some smoking, then I would look at them and think of that as a positive thing* (40+M, C2DE, Smoker)

One toxicity message (about formaldehyde) and one harm message (about long-term health risks) were thought to potentially discourage smokers from switching to e-cigarettes. The mention of formaldehyde gave some participants the impression that switching would not reduce exposure to harmful chemicals, while the unknown long-term health risks associated with e-cigarettes could be perceived as bad, or potentially worse, than known cigarette risks.

*Most tobacco smokers know that they* [cigarettes] *contain harmful chemicals, so fort hem it might just be like, “well, what difference does it make then?”* (18–39M, ABC1, Non-smoker)

*I think that would put me off as a smoker . . . the risks are there* [with cigarettes]*; you know what you’re going in for. But with that* [long-term health risks]*, that’s something completely unknown and you think, “oh”* (18–39F, C2DE, Smoker)

Regarding uptake, several groups expressed concerns that the relative risk messages may encourage nonsmokers to start using e-cigarettes due to the perception that they are less harmful than cigarettes.

*To a non-smoker, it’d be quite attractive thinking, “oh, it’s alright actually, these are quite healthy”* (40+F, C2DE, Dual user)

*If someone is really curious or something and they really want to try it, then in their heads they’re going to be like, “oh well, I’m not smoking, it’s not that bad; at least I’m doing this, it’s healthier”* (16–17M, ABC1)

Harm messages (on the health implications associated with e-cigarette use) and toxicity messages (about the chemicals present in e-cigarette products) were seen as potentially off-putting to nonsmokers due to the likely exposure to harmful toxins and the long-term impacts e-cigarettes may have on their health, with some viewing them as more effective than the current nicotine warning.

*I think it* [poisonous if swallowed] *has a bigger impact than the nicotine* [warning]*. I think if they were looking to buy a product, I think they’d steer clear of something because it’s got the word “poisonous” on it* (40+M, C2DE, Smoker)

*If you were a complete non-smoker and you just occasionally are getting an Elf Bar and then you got that message, ‘the long-term health risks are unknown’, I think that could still be a deterrent* (18–39M, ABC1, Non-smoker)

Two smokers commented that had the adolescent brain development message been on cigarette packs, they would have not started smoking.

*I didn’t actually know it could affect brain development, so I suppose it’s informative.That would have definitely put me off when I was young* (40+M, C2DE, Smoker)

*If I’d have read that . . . at 14, I probably would never have smoked* (18–39F, C2DE,Smoker)

### Alternative Pack Options

#### Strengthening Health Messaging

There was consensus that e-cigarette packaging should be regulated like cigarettes. Several participants felt that warnings should be more visible, taking prominence over promotional elements, and rotating, eg *“There’s space for usage of multiple messages, isn’t there? You’ll notice on every pack of cigarettes that you see, it’s a different one”* (18–39M, ABC1, Non-smoker). Options proposed for increasing visibility included increasing pack size and displaying messages on more pack surfaces. On-pack images depicting e-cigarette harms were also proposed; although some were unsure about image content, most felt that their presence would likely deter adolescents and nonsmokers from e-cigarettes.

*I think if it had the photos of the things, like it has on cigarette packages, that would deter. Because those things do actually scare me* (16–17M, ABC1)

#### Standardised Packaging and Devices

Without using the term “standardized”, several participants suggested that e-cigarette packaging should be plain, dull or monotone, in colors such as brown, gray or black, and without distinctive branding, eg “*Just do the same they do with cigarettes, plain across the board”* (F18–39, C2DE, Dual user). It was thought that this would make e-cigarettes less appealing to nonsmokers and younger users, discourage brand loyalty, and better target smokers switching to vapes.

*If, like, the packaging was dull, then they wouldn’t be drawn to it* (18–39M,C2DE, Dual user)

*The packaging and the flavours, it’s . . . very tempting . . . like that for non-smokers, it’s just so appealing, so they need to cut back on that. The packaging needs to be a bit more boring looking* (40+F, C2DE, Smoker)

Several participants suggested further possible measures to reduce the appeal of disposable e-cigarettes, particularly among youth consumers, including making devices less colorful, rather than according to flavor, and with no obvious brand features.

*These are like a collectable. And it’s, like, it’s a thing for kids to keep them and get all the colours and save them... If they were all black or grey, with no colours, nothing interesting on them, they wouldn’t be interested* (18–39F, C2DE, Smoker)

#### Warnings on e-Cigarettes

Several, primarily adolescent groups, proposed health messaging on e-cigarette devices, particularly disposables, given that they would not be discarded as quickly as the packaging.

*Maybe on the actual vape itself* (11–13F, C2DE)

*That might be better though, like, on the actual e-cig itself instead of the packaging, which you generally just throw away* (18–39M, C2DE, Dual user)

*Maybe if it had, like, ‘contains harmful chemicals’ and all that sort of stuff on the actual vape, cause you’re not going to throw away the actual vape when you’re vaping* (16–17F, C2DE)

It was suggested that having warnings on devices (eg disposables) would enhance warning salience as they would be more visible than on-pack warnings and seen each time a device was held or used, eg “*I feel like they’d actually look at the vape more than the pack*” (11–13F, C2DE). This increased visibility was thought to reinforce the message and extend its reach, which may influence how people think about vaping.

*Every time you’re smoking* [vaping] *it, you’re going to see it* (16–17F, C2DE)

*If that message was on anything else that you were putting near your mouth, you would take notice* (18–39M, C2DE, Smoker)

*They’d see it a lot more. Because, like, they’d probably have it in their hand quite a lot of the time, so they’d get to see it. So maybe it could change some people’s minds* (14–15F, C2DE)

## Discussion

Many participants were critical of the mandatory nicotine addiction warning,^7^ which failed to capture attention as it was not considered severe enough and was perceived as an afterthought or “tick-box” activity to meet legal requirements or for liability purposes, similar to alcohol packaging research^28^ and drawing comparisons to old cigarette pack warnings; tobacco industry sources acknowledge the importance of warnings in protecting companies from litigation.^36^ Although several participants noted that providing this information was necessary and helpful, it was not viewed as a deterrent, with some considering it common knowledge that nicotine is the addictive ingredient in e-cigarettes.

Regarding the alternative messages, the relative risk examples were considered potentially more helpful for smokers, more so than the nicotine addiction warning, supporting findings from a systematic review.^10^ However, many participants disagreed with the relative risk messages, arguing that there is no evidence that e-cigarettes are safe, particularly longer-term, highlighting that the messages do not advise people to use neither product and expressing concern about the potential unintended impact of encouraging youth or people who do not smoke to try e-cigarettes. These findings underscore the need for accurate messaging regarding relative and absolute product risks^37^; albeit, as scientific understanding of e-cigarettes continues to emerge, any potential warnings could contain uncertain language.^17^ While research suggests that smokers support positive messages about the benefits of smoking cessation,^38^ the e-cigarette wellness message displayed was considered unhelpful for those wanting to switch to a less harmful alternative.

The environmental harms of e-cigarettes is a growing concern,^39^ with approximately 1.3 million disposables discarded weekly in the United Kingdom,^40^ many containing non-biodegradable components.^39–41^ The litter messages displayed were typically dismissed due to the lack of clear instruction on how to dispose of e-cigarettes and refills correctly and were not considered conducive to behavior change, except potentially among some environmentally conscious people. Tobacco industry journals note that although manufacturers recognize the environmental harms of e-cigarettes, they do not have a long-term or feasible recycling strategy^41^; therefore, innovation aimed at reducing the environmental impact of these devices should be encouraged, with more environmentally friendly vapes emerging.^42^

Participants frequently referred to the design of cigarette packaging in the United Kingdom, which likely influenced responses, suggesting larger warnings and pictorial images, and alluding to standardized packaging to help deter youth from using e-cigarettes and encourage smokers to switch. While evaluations indicate that standardized tobacco packaging has improved health outcomes,^43^ few countries currently require standardized packaging for e-cigarette products^44^ (eg Israel, Denmark, and Finland), with others due for implementation^45,46^ (eg Australia and Norway) or considering doing so^47^ (eg Netherlands). Although few studies suggest that standardized e-liquid packaging may help reduce youth interest in trying products and increase health risk perceptions,^21^ with standardized e-cigarette packaging potentially making products less appealing to youth without similarly affecting adults.^22^ Nonetheless, the potential impact of standardized e-cigarette packaging on consumer behavior is limited to exploratory research given the absence of evaluative research in markets where this is required. In the United Kingdom, where e-cigarette products are considered a means of helping smokers quit, there is also a potential tension between the aim of standardized packaging (ie to reduce product appeal among youth and people who neither smoke nor vape) and the harm reduction aim of promoting e-cigarettes to smokers as a cessation aid. The United Kingdom Vaping Industry Association are opposed to standardized packaging, arguing that it may deter smokers from switching to e-cigarettes by conflating vaping with smoking and compounding misperceptions regarding their relative risks.^48,49^ Uncertainty regarding potential public health impacts of standardized e-cigarette packaging will persist without further research in the United Kingdom and elsewhere.

Several, primarily youth groups, proposed including health messaging on the e-cigarette device as it would increase visibility, being potentially seen every time it is held or used, thus reinforcing the message. The possibility of including a warning on cigarette sticks is attracting growing academic and policy attention, with research suggesting they reduce appeal, increase perceptions of harm, and reduce the likelihood of perceived trial.^50,51^ By the end of July 2024, Canada will be the first country to require warnings (eg “Cigarettes cause cancer”) on king-size cigarettes, followed by regular-size cigarettes, make-your-own cigarettes, and cigarillos thereafter.^52^ Dissuasive cigarettes, as they are often alluded to, are also being considered by the UK government.^53^ As participants frequently mentioned that e-cigarette packaging is discarded immediately, displaying a warning on e-cigarette devices would extend the messaging to the actual consumption experience.^54^ Additionally, reducing the appeal of e-cigarette devices warrants further consideration.^46^ Previous research shows that brightly colored e-cigarette devices are targeted at adolescents,^55^ with several participants in our study suggesting that having less colorful devices with restricted branding would help reduce product appeal, primarily to younger consumers.

Regarding limitations, although we included adolescents, adult nonsmokers, adult smokers, and adult smokers who also use e-cigarettes to learn from relevant populations, this led to a small number of groups for each sub-population, with some groups also having small participant numbers. Future studies may benefit from including people who use e-cigarettes but do not smoke to further explore the potential role of e-cigarettes in supporting smoking cessation and preventing relapse. The findings may have been influenced by socially desirable responses and the novelty of the alternative messages shown may also have influenced responses,^20^ with only a few minutes allocated to examining each message. Further research exploring responses to a range of messages is warranted,^23,30^ particularly given habituation,^56–58^ with few studies exploring the potential impact of warning images (eg depicting internal harm or people experiencing harms) on e-cigarette use.^11^ Additionally, the groups in England were not comoderated.

While it competes with companies’ efforts to make packs appealing, the health messaging on e-cigarette and refill packs can increase awareness of harms and help consumers make more informed decisions. Monitoring how e-cigarette manufacturers use e-cigarette packaging, as was the case for cigarette packaging in the United Kingdom, particularly prestandardized packaging,^59,60^ is important.

## Supplementary material

Supplementary material is available at *Nicotine and Tobacco Research* online.

ntae107_suppl_Supplementary_Material

## Data Availability

The data that support the findings of this study are available from the corresponding author, DJ, upon reasonable request.

## References

[1] Sontag JM , Wackowski OA, Hammond D. Baseline assessment of noticing e-cigarette health warnings among youth and young adults in the United States, Canada and England, and associations with harm perceptions, nicotine awareness and warning recall. Prev Med Rep. 2019;16:100966. doi: https://doi.org/10.1016/j.pmedr.2019.100966 31453077 PMC6704048

[2] Berry C , Burton S, Howlett E. The impact of e-cigarette addiction warnings and health-related claims on consumers’ risk beliefs and use intentions. J Public Policy Mark. 2017;36(1):54–69. doi: https://doi.org/10.1509/jppm.15.024

[3] Katz SJ , Lindgren B, Hatsukami D. E-cigarettes warning labels and modified risk statements: tests of messages to reduce recreational use. Tob Regul Sci. 2017;3(4):445–458. doi: https://doi.org/10.18001/TRS.3.4.6 30238022 PMC6141046

[4] Katz SJ , Shi W, Erkkinen M, Lindgren B, Hatsukami D. High school youth and E-cigarettes: the influence of modified risk statements and flavors on E-cigarette packaging. Am J Health Behav. 2020;44(2):130–145. doi: https://doi.org/10.5993/AJHB.44.2.2 32019647 PMC7266646

[5] Vogel EA , Tackett AP, Barrington-Trimis JL. Unclear labeling of nicotine products poses risks to consumers. Nicotine Tob Res. 2023;25(5):1057–1059. doi: https://doi.org/10.1093/ntr/ntac282 36511189 PMC10077932

[6] Wackowski OA , Hammond D, O’Connor RJ, Strasser AA, Delnevo CD. Considerations and future research directions for e-cigarette warnings—findings from expert interviews. Int J Environ Res Public Health. 2017;14(7):781. doi: https://doi.org/10.3390/ijerph14070781 28708124 PMC5551219

[7] UK Government. *The Tobacco and Related Products Regulations 2016*. legislation.gov.uk. Published 2016. Accessed August 18, 2023. https://www.legislation.gov.uk/uksi/2016/507/part/6/made

[8] Moore G , Brown R, Page N, et al Young people’s use of e-cigarettes in Wales, England and Scotland before and after introduction of EU Tobacco Products Directive regulations: a mixed-method natural experimental evaluation. Int J Drug Policy. 2020;85:102795. doi: https://doi.org/10.1016/j.drugpo.2020.102795 32854047 PMC7773804

[9] Kimber C , Frings D, Cox S, Albery IP, Dawkins L. Communicating the relative health risks of e-cigarettes: an online experimental study exploring the effects of a comparative health message versus the EU nicotine addiction warnings on smokers’ and non-smokers’ risk perceptions and behavioural intentions. Addict Behav. 2020;101:106177. doi: https://doi.org/10.1016/j.addbeh.2019.106177 31753541 PMC6891257

[10] Erku DA , Bauld L, Dawkins L, et al Does the content and source credibility of health and risk messages related to nicotine vaping products have an impact on harm perception and behavioural intentions? A systematic review. Addiction. 2021;116(12):3290–3303. doi: https://doi.org/10.1111/add.15473 33751707

[11] Lazard AJ , Ebrahimi Kalan M, Nicolla S, et al Optimising messages and images for e-cigarette warnings. Tob Control. 2023. doi: https://doi.org/10.1136/tc-2022-057859 PMC10733543 37344191

[12] Rohde JA , Noar SM, Sheldon JM, et al Identifying promising themes for adolescent vaping warnings: a national experiment. Nicotine Tob Res. 2022;24(9):1379–1385. doi: https://doi.org/10.1093/ntr/ntac093 35397474 PMC9356688

[13] Asfar T , Oluwole OJ, Pan Y, et al Youth exposure and response to the FDA health warning label on electronic cigarettes packaging: policy implications. Nicotine Tob Res. 2023;26(2):151–160. doi: https://doi.org/10.1093/ntr/ntad175 PMC10803120 37688562

[14] Ambrose BK , Rostron BL, Johnson SE, et al Perceptions of the relative harm of cigarettes and E-cigarettes among U.S. youth. Am J Prev Med. 2014;47(2 suppl 1):S53–S60. doi: https://doi.org/10.1016/j.amepre.2014.04.016 25044196 PMC4642861

[15] Cohn A , Johnson A, Abudayyeh H, King B, Wilhelm J. Pack modifications influence perceptions of menthol e-cigarettes. Tob Regul Sci. 2021;7(2):87–102.

[16] Avery RJ , Kalaji M, Niederdeppe J, et al Perceived threat and fear responses to e-cigarette warning label messages: results from 16 focus groups with U.S. youth and adults. PLoS One. 2023;18(6):e0286806. doi: https://doi.org/10.1371/journal.pone.0286806 37352255 PMC10289367

[17] Greiner Safi A , Kalaji M, Avery R, et al Examining perceptions of uncertain language in potential e-cigarette warning labels: results from 16 focus groups with adult tobacco users and youth. Health Commun. 2023;39(3):460–481. doi: https://doi.org/10.1080/10410236.2023.2170092 36717390 PMC10387126

[18] Moodie C , Angus K, Stead M. Consumer response to standardized tobacco packaging in the United Kingdom: a synthesis of evidence from two systematic reviews. Risk Manag Healthc Policy. 2021;14:1465–1480. doi: https://doi.org/10.2147/RMHP.S272259 33883953 PMC8053612

[19] Shankleman M , Sykes C, Mandeville KL, Di Costa S, Yarrow K. Standardised (plain) cigarette packaging increases attention to both text-based and graphical health warnings: Experimental evidence. Public Health. 2015;129(1):37–42. doi: https://doi.org/10.1016/j.puhe.2014.10.019 25542740 PMC4315810

[20] Babineau K , Clancy L. Young people’s perceptions of tobacco packaging: a comparison of EU Tobacco Products Directive & Ireland’s Standardisation of Tobacco Act. BMJ Open. 2015;5(6):e007352. doi: https://doi.org/10.1136/bmjopen-2014-007352 PMC4458627 26048206

[21] Simonavičius E , East K, Taylor E, et al Impact of e-liquid packaging on vaping product perceptions among youth in England, Canada, and the United States: a randomized online experiment. Nicotine Tob Res. 2024;26(3):370–379. doi: https://doi.org/10.1093/ntr/ntad144 37542732 PMC10882429

[22] Taylor E , Arnott D, Cheeseman H, et al Association of fully branded and standardized e-cigarette packaging with interest in trying products among youths and adults in Great Britain. JAMA Netw Open. 2023;6(3):e231799. doi: https://doi.org/10.1001/jamanetworkopen.2023.1799 36917111 PMC10015302

[23] Wackowski OA , Sontag JM, Hammond D, et al The impact of e-cigarette warnings, warning themes and inclusion of relative harm statements on young adults’ e-cigarette perceptions and use intentions. Int J Environ Res Public Health. 2019;16(2):184. doi: https://doi.org/10.3390/ijerph16020184 30634618 PMC6352031

[24] National Readership Survey. *Social Grade*. National Readership Survey. http://www.nrs.co.uk/nrs-print/lifestyle-and-classification-data/social-grade/. Accessed August 20, 2019.

[25] Office for National Statistics. *Deprivation and the Impact on Smoking Prevalence, England and Wales: 2017 to 2021*. Office for National Statistics. Published 2023. https://www.ons.gov.uk/peoplepopulationandcommunity/healthandsocialcare/drugusealcoholandsmoking/bulletins/deprivationandtheimpactonsmokingprevalenceenglandandwales/2017to2021. Accessed August 18, 2023.

[26] National Health Service. *Smoking, Drinking and Drug Use among Young People in England, 2021*; 2022. https://digital.nhs.uk/data-and-information/publications/statistical/smoking-drinking-and-drug-use-among-young-people-in-england/2021/part-1-smoking-prevalence-and-consumption. Accessed August 31, 2023.

[27] Jones D , Moodie C, Purves RI, Fitzgerald N, Crockett R. Alcohol packaging as a promotional tool: a focus group study with young adult drinkers in Scotland. J Stud Alcohol Drugs. 2022;83(4):565–573. doi: https://doi.org/10.15288/jsad.2022.83.565 35838434

[28] Jones D , Moodie C, Purves RI, Fitzgerald N, Crockett R. Health information, messaging and warnings on alcohol packaging: a focus group study with young adult drinkers in Scotland. Addict Res Theory. 2021;29(6):469–478. doi: https://doi.org/10.1080/16066359.2021.1884229

[29] Kimber C , Cox S, Frings D, Albery IP, Dawkins L. Development and testing of relative risk-based health messages for electronic cigarette products. Harm Reduct J. 2021;18(1):96. doi: https://doi.org/10.1186/s12954-021-00540-1 34496865 PMC8424813

[30] Brewer NT , Jeong M, Hall MG, et al Impact of e-cigarette health warnings on motivation to vape and smoke. Tob Control. 2019;28(e1):e64–e70. doi: https://doi.org/10.1136/tobaccocontrol-2018-054878 31292169 PMC6824616

[31] Patterson JG , Keller-Hamilton B, Wedel AV, Wagener TL, Stevens EM. Responses to e-cigarette health messages among young adult sexual minoritized women and nonbinary people assigned female at birth: assessing the influence of message theme and format. Drug Alcohol Depend. 2022;231:109249. doi: https://doi.org/10.1016/j.drugalcdep.2021.109249 35030509 PMC8815305

[32] Nott G. *Tobacco & Vaping 2022: Disposable Vapes Drive Stunning Growth* . The Grocer. Published 2022. https://www.thegrocer.co.uk/top-products/tobacco-and-vaping-2022-disposable-vapes-drive-stunning-growth/674498.article. Accessed August 18, 2023.

[33] Braun V , Clarke V. Using thematic analysis in psychology. Qual Res Psychol. 2006;3(2):77–101. doi: https://doi.org/10.1191/1478088706qp063oa

[34] Mitchell D , Moodie C, Ford A, et al Youth perceptions of brand variant names on standardised cigarette packs, and responses to replacing these with numbers: a focus group study in Britain. Drugs Educ Prev Policy. 2021;29(5):528–535. doi: https://doi.org/10.1080/09687637.2021.1902479 PMC9590399 36303721

[35] Mead EL , Cohen JE, Kennedy CE, Gallo J, Latkin CA. The role of theory-driven graphic warning labels in motivation to quit: a qualitative study on perceptions from low-income, urban smokers. BMC Public Health. 2015;15(1):92. doi: https://doi.org/10.1186/s12889-015-1438-6 25880277 PMC4349464

[36] Lambat I. *Have Governments Forgotten How to Dialogue?* Tobacco Asia. Published 2020. https://www.tobaccoasia.com/features/have-governments-forgotten-how-to-dialogue/. Accessed August 18, 2023.

[37] Bandi P , Asare S, Majmundar A, et al Relative harm perceptions of e-cigarettes versus cigarettes, U.S. adults, 2018–2020. Am J Prev Med. 2022;63(2):186–194. doi: https://doi.org/10.1016/j.amepre.2022.03.019 35868816

[38] Moodie C. Adult smokers’ perceptions of cigarette pack inserts promoting cessation: a focus group study. Tob Control. 2018;27(1):72–77. doi: https://doi.org/10.1136/tobaccocontrol-2016-053372 28153959

[39] Pourchez J , Mercier C, Forest V. From smoking to vaping: a new environmental threat? Lancet Respir Med. 2022;10(7):e63–e64. doi: https://doi.org/10.1016/S2213-2600(22)00187-4 35617988

[40] Material Focus. *One Million Single Use Vapes Thrown Away Every Week Contributing to the Growing e-Waste Challenge in the UK*. Material Focus. Published 2022. https://www.materialfocus.org.uk/press-releases/one-million-single-use-vapes-thrown-away-every-week-contributing-to-the-growing-e-waste-challenge-in-the-uk/. Accessed August 22, 2023.

[41] Tobacco China Online. *Pods Recovery: A “must-do” Issue for the Industry*. Tobacco Asia. Published 2022. https://www.tobaccoasia.com/features/pods-recovery-a-“must-do”-issue-for-the-industry/. Accessed August 22, 2023.

[42] AIRSCREAM UK. *AirsPops ONE USE Eco*. AIRSCREAM UK. Published 2023. Accessed August 22, 2023. https://corporate.airscreamuk.com/pages/one-use-eco

[43] Moodie C , Hoek J, Hammond D, et al Plain tobacco packaging: progress, challenges, learning and opportunities. Tob Control. 2022;31(2):263–271. doi: https://doi.org/10.1136/tobaccocontrol-2021-056559 35241599

[44] Tobacco Control Laws. *Find by Policy*. Tobacco Control Laws. https://www.tobaccocontrollaws.org/legislation/find-by-policy?policy=e-cigarettes&matrix=ecMainPolicies&handle=e-cigarettes&criteria=other-product-packaging-and-labeling-requirements&status=R. Accessed August 22, 2023.

[45] Nogrady B. Australia bans all vapes except on prescription to stem use in children. BMJ. 2023;381:1014. doi: https://doi.org/10.1136/bmj.p1014 37142274

[46] Scheffels J , Tokle R, Linnansaari A, Rasmussen SKB, Pisinger C. E-cigarette use in global digital youth culture. A qualitative study of the social practices and meaning of vaping among 15–20-year-olds in Denmark, Finland, and Norway. Int J Drug Policy. 2023;111:103928. doi: https://doi.org/10.1016/j.drugpo.2022.103928 36527908

[47] Vaping Post. *The Dutch Parliament to Consider Plain Packaging for Vapes*. Vaping Post. Published 2023. https://www.vapingpost.com/2023/03/14/the-dutch-parliament-to-consider-plain-packaging-for-vapes/ Accessed August 22, 2023.

[48] Harris D. *Banning Disposable Vapes a Bad Idea, Says Industry Association* . Talking Retail. Published 2023. https://www.talkingretail.com/news/industry-news/banning-disposable-vapes-a-bad-idea-says-industry-association-09-06-2023/. Accessed September 15, 2023.

[49] Spereall D. *Vaping Industry Hits Back Over Calls for Plain Packaging* . Telegraph & Argus. Published 2023. https://www.thetelegraphandargus.co.uk/news/23640403.vaping-industry-opposes-plain-packaging-councillors-concerns/. Accessed September 15, 2023.

[50] Moodie CS , Hiscock R, Thrasher J, Reid G. Perceptions of cigarette pack inserts promoting cessation and dissuasive cigarettes among young adult smokers in the UK: a cross-sectional online survey. BMJ Open. 2018;8(9):e019662. doi: https://doi.org/10.1136/bmjopen-2017-019662 PMC6129040 30185567

[51] Moodie C , Gendall P, Hoek J, et al The response of young adult smokers and nonsmokers in the United Kingdom to dissuasive cigarettes: an online survey. Nicotine Tob Res. 2019;21(2):227–233. doi: https://doi.org/10.1093/ntr/ntx261 29190398 PMC6329397

[52] Reynolds C. *Canada’s Warning Labels on Individual Cigarettes Now in Effect* . CTV News. Published 2023. https://www.ctvnews.ca/health/new-cigarette-warning-labels-in-effect-this-week-aim-to-deter-kids-convert-parents-1.6500532. Accessed August 22, 2023.

[53] UK Parliament. *Cigarette Stick Health Warnings Bill [HL]*. UK Parliament. Published 2021. https://hansard.parliament.uk/lords/2021-12-03/debates/81E90D02-1AD7-4F5C-ADD6-E703A5A045C3/CigaretteStickHealthWarningsBill(HL). Accessed September 15, 2023.

[54] Moodie C , O’Donnell R, Fleming J, et al Extending health messaging to the consumption experience: a focus group study exploring smokers’ perceptions of health warnings on cigarettes. Addict Res Theory. 2020;28(4):328–334. doi: https://doi.org/10.1080/16066359.2019.1653861 32939185 PMC7454525

[55] Smith MJ , MacKintosh AM, Ford A, Hilton S. Youth’s engagement and perceptions of disposable e-cigarettes: a UK focus group study. BMJ Open. 2023;13(3):e068466. doi: https://doi.org/10.1136/bmjopen-2022-068466 PMC10040067 36948552

[56] Argo JJ , Main KJ. Meta-analyses of the effectiveness of warning labels. J Public Policy Market. 2004;23(2):193–208. doi: https://doi.org/10.1509/jppm.23.2.193.51400

[57] Li L , Borland R, Yong H, et al Longer term impact of cigarette package warnings in Australia compared with the United Kingdom and Canada. Health Educ Res. 2015;30(1):67–80. doi: https://doi.org/10.1093/her/cyu074 25492056 PMC4296892

[58] Woelbert E , D’Hombres B. Pictorial health warnings and wear-out effects: evidence from a web experiment in 10 European countries. Tob Control. 2019;28(e1):e71–e76. doi: https://doi.org/10.1136/tobaccocontrol-2018-054402 30610081 PMC6824613

[59] Moodie C , Hastings G. Tobacco packaging as promotion. Tob Control. 2010;19(2):168–170. doi: https://doi.org/10.1136/tc.2009.033449 20378594

[60] Moodie C , Angus K, Mitchell D, Critchlow N. How tobacco companies in the United Kingdom prepared for, and responded to, standardised packaging of cigarettes and rolling tobacco. Tob Control. 2018;27(e1):e85–e92. doi: https://doi.org/10.1136/tobaccocontrol-2017-054011 29321273

