# Supplementary material for: The Role of e-Cigarette Packaging as a Health Communications Tool: A Focus Group Study With Adolescents and Adults in England and Scotland
Source: Nicotine Tob Res. 2024 Jun 6;27(4):705–13. doi: 10.1093/ntr/ntae107 (PMC11931217; doi:10.1093/ntr/ntae107)
Supplement: ntae107_suppl_Supplementary_Material [file ntae107_suppl_supplementary_material.docx]

**Perceptions of e-cigarette packaging messaging** (Version 5: 05/09/2022)

**PART A - Perceptions of current messaging:**

• Thinking about the packaging of these products, what type of information do you think needs to be on e-cigarette packaging? (prompt with other products they use if they’re struggling)

Now I’d like you to think about the messages and information on e-cigarette packaging, including text information and symbols.

PICK UP GEEK BAR WITH VARIOUS INFO/SYMBOLS, SHOW THEM WHAT YOU MEAN, ESPECIALLY SYMBOLS AS THESE MAY HAVE NOT BEEN NOTICED.

**Activity**: Get participants to each pick up a pack and take a look at it, to see what messages and symbols they see on each. Go round group and ask participants to describe what is on their pack.

- Opportunity to get them to discuss how similar or different the packs are in terms of use of messages and symbols. Probe for understanding of what the messages and symbols mean, salience, ease of reading, and credibility/believability.
- How often do you read or look closely at the messaging and warnings on or inside e-cigarette product packaging?

SHOW ANY PACK (i.e. mandatory warning) AND SHEET with the text of the nicotine message ‘This product contains nicotine which is a highly addictive substance’.

- What do you think of when you see this message?
- What do you understand about nicotine? In what products do you find it? Is nicotine harmful to health?
- Who do you think this message is targeted at?
- Do you think people pay attention to a message like this?
- What do you think is the purpose/point of this message?

SHOW PACK (Geek Bar) AND SHEET with the text ‘This product is not for use by children and non-smokers’.

- What thoughts, if any, do you have when you see this message?
- When you see the word ‘children’, what age range do you think this covers?
- Do you think children and non-smokers would pay attention to a message like this?
- What do you think is the purpose/point of this message?

Another message on or in packs explains that there are possible adverse effects of e-cigarette products, including toxicity: SHOW FANTASI BAR (side) & SHEET with the text ‘WARNING: Toxic if swallowed or in contact with skin'

- What do you think of when you see this message?
- Does it capture attention? (Why? Design)
- Is it informative? (Why?)
- What do you think is the purpose/point of this message?

**PART B - Alternative messaging (Harm, Relative risk , Chemicals, Litter, Wellness):**

I would now like to consider other types of messaging that could appear on e-cigarette product packaging. Again, **please do not open any of them** as they’ll be used in other group discussions.

RANDOMISE SHOWING PACKS, but keep in 5 themes/5 sets – 1 set at a time

- What do you think of when you see this message? (Why?)
- Who do you think this message is targeted at?
- Do you think people pay attention to a message like this?
- What do you think is the purpose/point of this message
- Do you think this message would encourage or discourage **smokers** from using e-cigarettes, or would it not make a difference? (prompt if needed)
- Would it encourage or discourage **non-smokers** from using e-cigarettes, or would it not make a difference? (prompt if needed)

• Can you think of any other ways that the messages on e-cigarette products could be changed to make them more noticeable?

**PART C - Alternative pack options:**

This is the last section, just a couple more questions.

- Aside from warnings, can you think of any other ways that the packaging of e-cigarette products could be changed to make them **less appealing to non-smokers**? (*if they mention pictorials, what might these look like?)*
- Similarly, can you think of any other ways that the packaging of e-cigarette products could be changed to make them **more appealing to smokers**?
- Those last two questions capture what governments seem to be interested in, i.e. encouraging smokers to use these products, while discouraging non-smokers from using them. Before we end, is there anything else you would like to share?

**STOP RECORDING**

**Debrief & incentives:**

Before we end I would like to explain the purpose of our discussions today. We are interested in your thoughts and opinions on e-cigarette packaging and messaging, and whether alternative messaging may help encourage smokers to switch to these products and discourage non-smokers from using them, so thank you for your time and your input. While current evidence suggests that e-cigarettes are less harmful than smoking cigarettes, and therefore a safer alternative than cigarettes for existing smokers, they can result in addiction to nicotine, they are not risk-free, and the long-term health effects are unknown; therefore, non-smokers should not be encouraged to use these.

I realise by showing examples of e-cigarette related health messaging this may have caused you some discomfort and I apologise if this has been the case. Before we finish, please let me know if you have any concerns about the study or questions you would like to ask me. If not, once again, thank you for your participation.

*Distribute envelopes with incentives and list of websites.*
